# RNA-Binding Motif Protein 11 (RBM11) Serves as a Prognostic Biomarker and Promotes Ovarian Cancer Progression

**DOI:** 10.1155/2021/3037337

**Published:** 2021-08-14

**Authors:** Chunhong Fu, Ming Yuan, Jie Sun, Gang Liu, Xiaojuan Zhao, Wei Chang, Zhongling Ma

**Affiliations:** ^1^Department of Obstetrics & Gynecology, Inner Mongolia People's Hospital, Inner Mongolia 010017, China; ^2^Department of Oncology, Chang'an District Hospital, The First Affiliated Hospital of Xi'an Jiaotong University, Xi'an, Shanxi Province 710100, China; ^3^Shenzhen International Travel Health Care Center, Shenzhen Customs District Port Outpatient Clinics, Shenzhen Customs District, Shenzhen 518033, China

## Abstract

Ovarian cancer is one of the most lethal gynecologic malignancies for women. Due to the lack of efficient target therapy, the overall survival rate for patients with advanced ovarian cancer is still low. Illustrating the molecular mechanisms dictating ovarian cancer progression is critically important to develop novel therapeutic agents. Here, we found that RNA-binding motif protein 11 (RBM11) was highly elevated in ovarian cancer tissues compared with normal ovary, while RBM11 depletion in ovarian cancer cells resulted in impaired cell growth and invasion. Moreover, knockdown of RBM11 also retarded tumor growth in the A2780 ovarian cancer xenograft model. Mechanically, we found that RBM11 positively regulated Akt/mTOR signaling pathway activation in ovarian cancer cells. Thus, these results identify RBM11 is a novel oncogenic protein and prognostic biomarker for ovarian cancers.

## 1. Introduction

Ovarian cancer is one of the leading causes of death for gynecologic tumors among women, and more than 300,000 new ovarian cancer cases were diagnosed each year [1]. Although the rapid progression in cancer research studies, the five-year survival rate for ovarian cancer is still low (<47%) due to its fast progression and frequent recurrence [2, 3], which demands novel therapeutic regimens. Understanding the mechanism for ovarian progression and identifying novel molecular switch that dictate ovarian cancer malignancy are critically important to develop novel treatments for this deadly disease.

RNA-binding proteins (RBPs) are proteins that bind to RNA molecules and participate in posttranscriptional control of RNA functions [4–6]. RBPs play important roles in various cellular processes, such as RNA splicing, RNA stabilization, RNA transportation, RNA localization, and RNA modification [7, 8]. More than 2000 proteins have been identified to interact with RNA transcripts and are recognized as RBPs. RBPs are involved in various physiological and pathological processes including development, metabolism, proliferation, pluripotency, tumors, and immunity [9–11]. Recently, the functions of RBPs in cancers have been extensively studied [6]. RBPs could exert either oncogenic or tumor suppression roles in different human cancers [12]. For instance, RNA-binding protein Nova1 and NELFE promote cancer growth in hepatocellular carcinoma (HCC) cells [13, 14], whereas RBM47 and RBM43 have been shown to suppress HCC growth [15, 16].

RNA-binding motif protein 11 (RBM11) is a RNA splicing factor containing an RNA Recognition Motif (RRM) at the amino terminus (N-terminal), and its expression has been observed in multiple normal human tissues, including the brain, testis, and spleen [17]. Although the physiological function of RBM11 has not been clearly defined, it is putatively linked to Down syndrome due to its chromosome 21 location [17]. Recently, upregulation of RBM11 protein level has been observed in glioblastoma and RBM11 could promote glioblastoma cell proliferation and invasion *in vitro* and *in vivo* [18]. Nonetheless, the oncogenic roles of RBM11 in other cancer types beyond glioblastoma are needed to be further investigated. Here, we studied the functions of RBM11 in ovarian cancer and found that RBM11 was overexpressed in ovarian cancer tissues and exerts oncogenic roles in ovarian cancer through activating Akt/mTOR signaling. Thus, these results provide evidences that RBM11 is an oncogenic protein in cancers.

## 2. Materials and Methods

### 2.1. Cell Culture and Transfection

Human ovarian cancer cells A2780 and OVCAR-3 were obtained from the American Type Culture Collection (ATCC) and cultured in the Roswell Park Memorial Institute- (RPMI-) 1640 medium supplemented with 10% fetal bovine serum (FBS; Hyclone). Cells were tested for mycoplasma contamination every two months. Cells were transfected with plasmid DNA using lipofectamine 3000 reagent (Invitrogen, Carlsbad, CA, USA) according to the manufacturer's instruction.

### 2.2. Plasmids and Antibodies

RBM11 and control shRNAs were purchased from Sigma-Aldrich (St. Louis, MO, USA), and their targeting sequences were as follows: RBM11 shRNA-1,5′-GTT CCG AAA GTC TAA GAA GAA-3′; RBM11 shRNA-2,5′-CCC AGC TCA TAT AAA TGG ACT-3′. The flag-RBM11 plasmid was purchased from Sino Biological Inc. (Beijing, China). Anti-RBM11 (17220-1-AP) antibody was purchased from Proteintech (Wuhan, China), and anti-pAkt S473 (#9271), anti-Akt (#4691), anti-pmTOR S2448 (#5536), and anti-mTOR (#2972) antibodies were purchased from Cell Signaling Technology (Beverly, MA, USA). Anti-*β*-actin (sc-47778) and anti-flag (sc-166355) antibodies were purchased from Santa Cruz Biotechnology (Santa Cruz, CA, USA).

### 2.3. Establishment of RBM11 Stable Knockdown Cells

RBM11 shRNA and lentiviral packaging vectors (psPAX2 and pCMV-VSV-G) were cotransfected into 293FT cells by lipofectamine 3000 transfection reagent. 72 hours after transfection, virus-containing medium supernatant was harvested by centrifugation. OVCAR-3 and A2780 cells were infected with lentivirus in the presence of 10 *μ*g/ml of polybrene. After selection by puromycin, RBM11 knockdown efficacy was confirmed by western blot.

### 2.4. Colony Formation Assay

Cells expressing control or RBM11 shRNA were seeded in a 6-well plate at a density of 1 × 10^3^ cells per well. Cells were grown for 10 days; then, the clones were fixed and stained with 0.5% crystal violet in 20% methanol followed by counting the number of colonies. Relative colony formation was expressed as a percentage normalized to cells expressing control shRNA.

### 2.5. Transwell Invasion Assay

The upper chambers of transwell inserts (Corning, NY, USA) were coated with matrigel (1 : 10 dilution) (Invitrogen, Carlsbad, CA, USA) at 37°C for 1 hour. Cells were starved for 24 hours before harvesting and suspending at 1 × 10^5^/ml in a serum-free medium. Then, 200 *μ*l of cell suspension was seeded into a matrigel-coated upper chamber of inserts. The bottom chamber of inserts was placed into a 24-well plate with a 500 *μ*l medium containing 10% FBS. After culturing at 37°C for 24 h, the transwell inserted was removed, cells on the upper membrane were wiped using a cotton swap, and the cells on the bottom chamber were then stained with crystal violet in 20% methanol and counted.

### 2.6. Xenograft Tumor Growth

The cancer xenograft was established as previously described [19]. Briefly, 4-6 weeks of female nude mice were purchased from GemPharmatech (Nanjing, China). All the animal protocols were approved by the Animal Care and Use Committee of the First Affiliated Hospital of Xi'an Jiaotong University. A2780 xenograft was performed as previously reported [20]; briefly, 1 × 10^7^ of A2780 cells expressing control or RBM11 shRNA were subcutaneously implanted into mouse flanks. Tumor growth was monitored, the tumor length (*L*) and width (*W*) were measured every five days, and tumor volume (*V*) was calculated by formula *V* = (*W*^2^ × *L*)/2.

### 2.7. Immunohistochemistry Assay

The ovarian cancer tissues and adjacent normal ovarian tissues were purchased from Alenabio Co., Ltd. (Xi'an, China). The tumor sections were firstly deparaffinized with 100% xylene, followed by rehydration using gradient ethanol (100%, 95%, 70%, 30%, and 0%). After inactivation of endogenous peroxidase by 3% H_2_O_2_ and heat-induced retrieval antigen, IHC staining was performed using the R.T.U Vectastain Kit (Vector Laboratories, Burlingame, CA) according to the manufacturer's instructions. The Ki67 (abcam, ab15580) antibody was diluted at 1 : 500. The percentage of Ki67-positive cells was determined from three separate fields.

### 2.8. Western Blot Assay

Western blot was performed as previously described. Briefly, proteins in cell lysates were resolved on SDS-PAGE and were transferred on PVDF membrane after electrophoresis. After blocking in 5% milk in PBS, the membrane was incubated with primary antibody and second antibody sequentially, following band detection by enhanced chemiluminescence (ECL) using Pierce™ ECL Western Blotting Substrate (GE Healthcare Bio-Sciences).

### 2.9. Statistical Analysis

A two-sided unpaired Student's *t*-test was used to calculate the statistical significance of differences. Results were considered statistically significant at *p* < 0.05. All data are presented as mean ± standard deviation (S.D.) from at least three replicates.

## 3. Results

### 3.1. RBM11 Is Overexpressed in Ovarian Cancer Tissues

**Figure 1:**
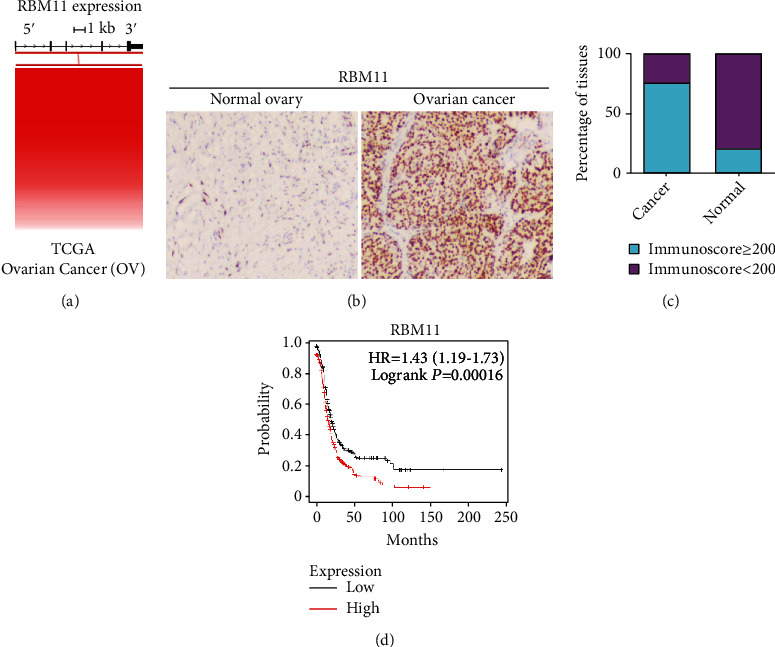
RBM11 is overexpressed in ovarian cancer tissues and is highly associated with poor survival in ovarian cancer patients. (a) RBM11 mRNA expression in ovarian cancer tissues was assessed with TCGA database. (b, c) RRM1 protein levels in normal ovary and ovarian cancer tissue were analyzed by Immunohistochemistry (IHC) staining. Representative image of staining (b) and quantitative analysis of immunoscore (c) were provided. (d) Overall survival assay in ovarian cancer patients with high RBM11 mRNA level and low RBM11 mRNA level was performed using the Kaplan-Meier plotter online server (https://www.kmplot.com/analysis).

RBM11 has been shown to exert oncogenic roles in glioblastoma cells [18]; to examine its function in ovarian cancers, we first examined RBM11 mRNA expression and its gene copy numbers in ovarian cancer tissues from the Cancer Genome Atlas (TCGA) database and found that although RBM11 copy number was not frequently amplified, RBM11 mRNA was highly elevated in ovarian cancer tissue (Figure 1(a) and Supplementary Figure 1). To further validate the RBM11 protein levels in ovarian cancer tissues, we performed Immunohistochemistry (IHC) staining and the result (Figures 1(b) and 1(c)) showed that RBM11 protein level was significantly overexpressed in ovarian cancer tissues compared with normal ovary. In addition, a high level of RBM11 was associated with poor survival in ovarian cancer patients (Figure 1(d)).

### 3.2. RBM11 Promotes Ovarian Cancer Cell Growth

**Figure 2:**
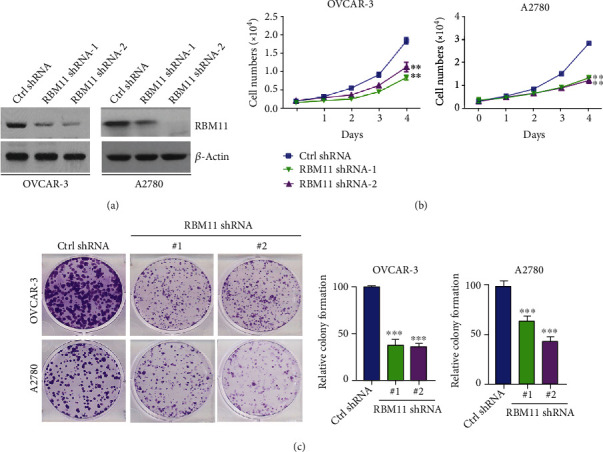
Knockdown of RBM11 inhibits ovarian cancer cell growth. (a) Western blot analysis of RBM11 protein level in OVCAR-3 and A2780 cells stably transfected with control (ctrl), RMB11 shRNA-1, or RBM11 shRNA-2. (b) Cell growth curve of OVCAR-3 and A2780 cells expressing ctrl shRNA, RBM11 shRNA-1, or RBM11 shRNA-2. (c) Colony formation assay of cells expressing ctrl shRNA or RBM11 shRNA. Left, the representative colony growth; right, the relative quantification of colony number. ^∗∗^*p* < 0.01 and ^∗∗∗^*p* < 0.001, by 2-tailed *t*-test, when compared with cells expressing ctrl shRNA.

To test the role of RBM11 in ovarian cancer progression, we knocked down RBM11 expression in ovarian cancer cells including OVCAR-3 and A2780 using two distinct shRNA targeting the RBM11 coding region (Figure 2(a)). Interestingly, we found that silence of RBM11 significantly inhibited growth in both OVCAR-3 and A2780 cells (Figure 2(b)). Moreover, clonogenic formation assay also further revealed that depletion of RBM11 significantly reduced colony formation capacity in OVCAR-3 and A2780 cells (Figure 2(c)). These results demonstrated that RBM11 is required for ovarian cancer cell proliferation *in vitro*.

### 3.3. RBM11 Enhances Ovarian Cancer Invasion

**Figure 3:**
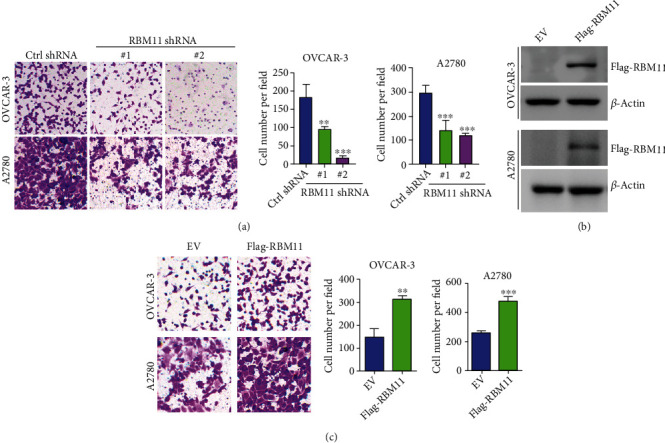
RBM11 promotes ovarian cancer invasion. (a) Transwell invasion analysis of OVCAR-3 and A2780 cells expressing ctrl or RBM11 shRNA. Left, representative staining of cells on the bottom membrane of the transwell insert was provided; right, cells on the bottom membrane per field were quantified. (b) Flag-tagged RBM11 was stably expressed in OVCAR-3 or A2780 cells; the expression of flag-RBM11 was examined by western blot. (c) Transwell analysis of OVCAR-3 or A2780 cells expressing flag-RBM11. Representative image (left) and quantitative analysis (right) were shown. ^∗∗^*p* < 0.01 and ^∗∗∗^*p* < 0.001, by 2-tailed *t*-test, when compared with cells expressing ctrl shRNA.

Beyond proliferation, the invasion capacity of cancer cells is also an important factor, affecting the prognosis of cancer patients. Therefore, we performed a transwell assay to evaluate the role of RBM11 in ovarian cancer invasion, as shown in Figure 3(a); the number of invaded OVCAR-3 and A2780 cells expressing RBM11 shRNA is remarkable less than that expressing control (ctrl) shRNA. To further confirm the effect of RBM11 on ovarian cell invasion, we ectopically expressed flag tagged RBM11 into OVCAR-3 and A2780 cells (Figure 3(b)). Similarly, overexpression of flag-RBM11 significantly enhanced cell invasion (Figure 3(c)). Thus, these results indicated that RBM11 is also essential for ovarian cancer invasion and metastasis.

### 3.4. RBM11 Enhances Akt/mTOR Activation in Ovarian Cancer Cells

**Figure 4:**
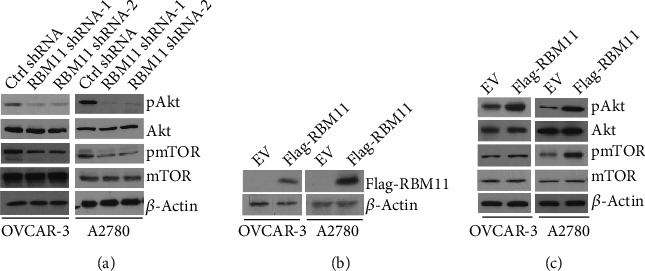
RBM11 promotes Akt/mTOR activation in ovarian cancer cells. (a) Western blot analysis of pAkt (S473), Akt, pmTOR (S2448), and mTOR levels in cells expressing control (ctrl) or RBM11 shRNA. (b) Flag-tagged RBM11 was transfected into OVCAR-3 and A2780 cells, and the expression of flag-RBM11 was validated by western blot assay. (c) Western blot analysis of indicated protein levels in cells transfected with empty vector (EV) or flag-RBM11.

As one of the most frequently activated signaling pathways in ovarian cancer cells, Akt plays critical roles in ovarian cancer proliferation and invasion [21]. Akt is commonly activated by phosphatidylinositol 3 kinase (PI3K) and activates the downstream signal through mTOR to promote translation of target genes involved in cell proliferation and invasion [22]. To explore the molecular mechanism by which RBM11 exerts its oncogenic function in ovarian cancer cells, we tested whether RBM11 could affect Akt signaling. As shown in Figure 4(a), we detected a significant decrease of Akt phosphorylation and mTOR phosphorylation in cells expressing RBM11 shRNA, which suggests that RBM11 might positively regulate Akt signaling activation. To further confirm the role of RBM11 in Akt signaling, exogenous RBM11 was ectopically expressed in OVCAR-3 and A2780 cells (Figure 4(b)). Consistent with RBM11 knockdown, overexpression of RBM11 resulted in an increase of phosphorylation of Akt and phosphorylation of mTOR (Figure 4(c)). These results suggest that RBM11 promotes ovarian cancer progression through stimulating Akt/mTOR signaling pathways.

### 3.5. RBM11 Knockdown Retards Ovarian Cancer Growth *In Vivo*

**Figure 5:**
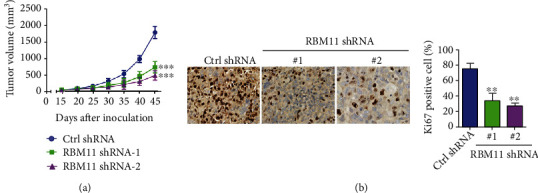
RBM11 silence retarded tumor growth in the A2780 xenograft model. (a) A2780 cells expressing control (ctrl) or RBM11 shRNA were implanted into nude mice, the tumor volumes were measured, and the tumor growth curve was shown. (b) Immunohistochemistry (IHC) analysis of Ki67 levels in tumor tissue. ^∗^*p* < 0.01 and ^∗∗∗^*p* < 0.001, by 2-tailed *t*-test, when compared with cells expressing ctrl shRNA.

To validate the oncogenic roles of RBM11 in ovarian cancer *in vivo*, we constructed a xenograft model using A2780 cells expressing control (ctrl) or RBM11 shRNA. Consistent with *in vitro* results, we found that knockdown of RBM11 significantly inhibited tumor growth in the A2780 xenograft model (Figure 5(a)). Meanwhile, A2780 xenograft tumors with RBM11 knockdown contained significantly less Ki67 protein level, a well-defined proliferation marker, compared with tumors expressing control shRNA (Figure 5(b)). Thus, these results demonstrated that RBM11 promotes ovarian cancer growth *in vivo*.

## 4. Discussion

Ovarian cancer is a leading cause of death from gynecological malignancies. Although the prognosis is relatively favorable if diagnosed at an early stage, the 5-year survival rates are only 30-40% for most ovarian cancer patients when they are detected at an advanced stage [1]. Currently, surgery followed by traditional chemotherapy including carboplatin and paclitaxel is employed as the first-line treatment for patients with high-grade epithelial ovarian cancer [3]. Beyond chemotherapy, PARP inhibitors such as olaparib and rucaparib showed favorable outcomes during the maintenance therapy for patients with advanced ovarian cancer [23]; however, PARP inhibitors are only approved and effective for patients with BRCA1/2 mutation, which accounts for less than 40% of ovarian cancer cases [24]. Therefore, it is an urgent need to develop novel targeted therapies for patients with high-grade ovarian cancers. In the present study, we identified that RBM11 is especially expressed in ovarian cancer tissues, but not in normal ovary tissue, which demonstrates that RBM11 is a feasible target for drug design in future ovarian cancer treatment. However, the functional domain of RBM11 used as the drug interacting pocket needed to be further identified.

Regulation of RNA metabolism is important to maintain normal cellular and physiological circumstances. RNA-binding proteins (RBPs) are involved in posttranscriptional control of transcriptome via alternative splicing [25], RNA modification [26], and RNA stability [4]. Accumulating evidences indicate that dysregulation of RNA metabolism resulted from abnormal RBP function plays critical roles in development [27], immunity [9], and neural function [28], as well as cancer progression [9, 12, 23]. However, the function of this group of proteins in ovarian cancer has rarely been reported. In this study, we discovered a novel mechanism governing ovarian cancer progression through RBM11. RBM11 enhances ovarian cancer proliferation and invasion *in vitro* and *in vivo*, which highlighted the importance of RBPs in ovarian cancer progression.

Among the members of RNA-binding protein, the biochemical and physiological function of RBM11 has not been well defined. It is previously reported that RBM11 protein was upregulated in glioblastoma tissues and promoted glioblastoma cell progression [18]; however, the underlying mechanism for RBM11's oncogenic roles in cancer cells has not been defined. In our present study, we investigated the function of RBM11 in ovarian cancers. In agreement with the glioblastoma study, we also found that RBM11 was highly elevated in ovarian cancer tissues and positively regulated ovarian cancer growth and invasion. Furthermore, we demonstrated that RBM11 affected the Akt/mTOR signaling pathway, which provides evidence and explanation why RBM11 could promote cancer cell progression.

## 5. Conclusion

RBM11 is elevated in ovarian cancer tissues and promotes ovarian cancer growth and invasion through activating the Akt/mTOR signaling pathway. Our finding has identified a functional role of RBPs in ovarian cancer progression and provided a novel molecular target for ovarian cancer therapy.

## Data Availability

All data used to support the findings of this study are included within the article and are available upon request from the corresponding author.

## Supplementary Materials

Supplementary Materials Supplementary Figure S1: RBM11 copy number in TCGA ovarian cancer database was analyzed by UCSC Xena online software (https://xena.ucsc.edu/).
