# Supplementary material for: RNA-Binding Motif Protein 11 (RBM11) Serves as a Prognostic Biomarker and Promotes Ovarian Cancer Progression
Source: Dis Markers. 2021 Aug 14;2021:3037337. doi: 10.1155/2021/3037337 (PMC8382552; doi:10.1155/2021/3037337)
Supplement: Supplementary Materials — Supplementary Figure S1: RBM11 copy number in TCGA ovarian cancer database was analyzed by UCSC Xena online software (https://xena.ucsc.edu/). [file 3037337.f1.docx]

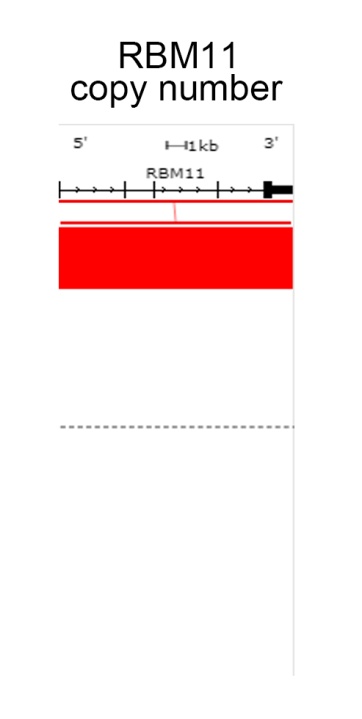


**Supplementary Figure S1**. RBM11 copy number in TCGA ovarian cancer database was analyzed by UCSC Xena online software(https://xena.ucsc.edu/)
